# Strategies for picking membrane-associated particles within subtomogram averaging workflows†

**DOI:** 10.1039/d2fd00022a

**Published:** 2022-04-12

**Authors:** Euan Pyle, Joshua Hutchings, Giulia Zanetti

**Affiliations:** a Institute of Structural and Molecular Biology, Birkbeck College Malet St. London WC1E 7HX UK g.zanetti@bbk.ac.uk; b Division of Biological Sciences, University of California San Diego La Jolla CA USA

## Abstract

Cryo-electron tomography (cryo-ET) with subtomogram averaging (STA) has emerged as a key tool for determining macromolecular structure(s) *in vitro* and *in situ*. However, processing cryo-ET data with STA currently requires significant user expertise. Recent efforts have streamlined several steps in STA workflows; however, particle picking remains a time-consuming bottleneck for many projects and requires considerable user input. Here, we present several strategies for the time-efficient and accurate picking of membrane-associated particles using the COPII inner coat as a case study. We also discuss a range of particle cleaning solutions to remove both poor quality and false-positive particles from STA datasets. We provide a step-by-step guide and the necessary scripts for users to independently carry out the particle picking and cleaning strategies discussed.

## Abstract 2

In cryo-tomography and subtomogram averaging, particle picking constitutes a major bottleneck and requires significant user time and expertise. Here we summarise particle-picking strategies, focusing in particular on membrane-associated particles.

## Graphical abstract

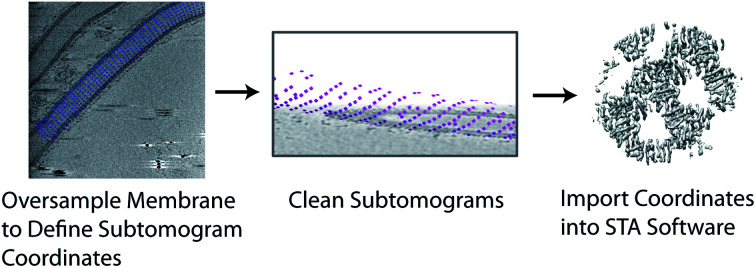

## Introduction

Many biological events are organised and orchestrated around membranes. The molecular understanding of such processes requires the structural determination of membrane-associated proteins in the presence of a lipid bilayer, whether through *in vitro* reconstitution approaches or *in situ*.

Cryo-electron tomography (cryo-ET) with subtomogram averaging (STA) has emerged as a key tool for obtaining high-quality structures of various classes of proteins, including membrane-associated protein complexes.^1–5^ In recent years, a significant effort has been put into automating many of the steps in cryo-ET and STA, but particle picking remains a challenge, as systems under study differ widely.

An exemplary case study is that of the COPII coat, which assembles around membranes to induce curvature and forms membrane carriers of a variety of morphologies, including spherical vesicles, tubules, and irregular shapes.^6–8^ The COPII coat consists of an inner coat, which forms a tightly packed and regularly arranged lattice very close to membranes, and the outer coat, which consists of long rods arranged into cage-like structures further away from the membrane. Understanding how the COPII coat can remodel membranes in a wide-range of curvatures and morphologies requires understanding the coat architecture in its various forms.

**Fig. 1:**
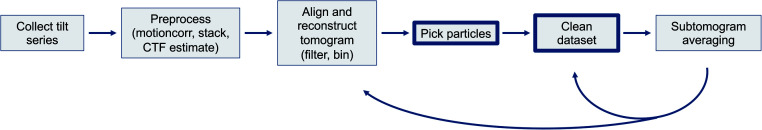
A schematic workflow of the first stages of typical subtomogram averaging projects. The focus of this article is highlighted (particle picking and dataset cleaning).

In our lab, we use cryo-ET and STA to study the COPII coat structure assembled on membranes *in vitro*, and have developed and optimised pipelines for data processing of the COPII coat on a variety of membrane morphologies.^6–8^ Here, we discuss our general workflow, with a specific emphasis on describing and detailing our particle picking strategies for different membrane shapes (Fig. 1). Our processing workflow, and more specifically our particle picking strategies, can serve as a model for many tightly-packed membrane-associated proteins.

## Workflow

### General guide to pre-processing raw cryo-ET data and tomogram generation

The first step in a typical STA workflow is pre-processing the raw images from the electron microscope. Cryo-ET data is collected as a series of images of a sample tilted over a range of angles (most frequently ±60°), known as a tilt-series. Initially, movies of raw images from each angle within the tilt-series must be motion corrected to account for specimen movement during data collection. For motion correction, software packages such as MotionCor2 can be used.^9^ A tilt-series stack containing each summed motion-corrected image ordered from low to high tilt (or *vice versa*) is then generated by an image stacking program such as the newstack function within IMOD.^10^ To correct for inaccuracies in image tracking during data collection, the tilt series is then aligned either with or without the aid of fiducial markers. This process can be carried out automatically using software packages such as Dynamo or IMOD.^11–13^ The contrast transfer function (CTF) of each tilt image can be estimated using programs such as CTFFIND4.^14^ Tomograms are then commonly reconstructed by back-projecting the individual tilt images to form a 3D-volume using a weighted back-projection algorithm.^15^ During tomogram reconstruction, one can optionally carry out CTF correction and/or dose-weighting, or leave these steps for later during STA.^16,17^ For direct interpretation, tomograms are typically binned, which assists in the identification of particles and other objects. An extensive analysis of pre-processing and tomogram generation strategies has been covered by recent reviews.^3–5^

### Tomogram denoising and mitigation of missing-wedge artifacts

Tomograms generated in cryo-ET suffer from two major issues: a low signal to noise ratio (SNR) and the ‘missing wedge’ problem.^5^ The SNR of cryo-tomograms can be very low due to the need to use a relatively low electron dose to avoid excessive radiation damage. Moreover, the tilting of the sample increases the thickness of the ice the electron beam passes through, which in turn reduces image contrast. The missing wedge problem arises from the fact that with current set-ups it is only possible to tilt the sample a maximum of ±60° before the ice becomes too thick and the contrast of the image too low to extract meaningful information. Consequently, there are a number of missing views during back-projection of tilt images to reconstruct the tomograms, which causes resolution anisotropy. This manifests as a smearing effect along the direction of the electron beam. The low SNR and the missing wedge effect make many steps in the STA workflow, including particle picking, challenging as it is difficult to accurately identify and align particles of interest.

**Fig. 2:**
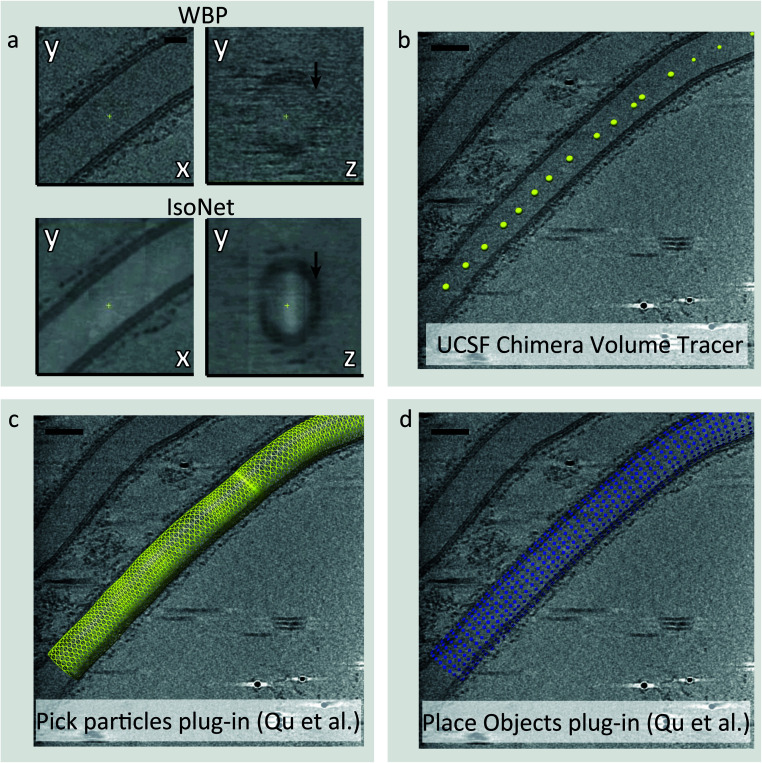
Picking particles from tubular membranes. (a) Comparison of a COPII coated tube after standard weighted back projection (top) and after filtering with IsoNet (bottom). In addition to the increased contrast, IsoNet improves isotropy, rendering membranes equally visible on the *XY* plane (left) and along the *Z* axis (right, black arrows). (b) Tube axes are picked using UCSF Chimera volume tracer tool, and saved individually as .cmm files. (c) The Pick Particle Chimera plugin^20^ is used to load the picked tube axis coordinates and set the diameter of the tube. At this stage, the sampling of subtomograms to be extracted is also defined on the membrane surface. (d) The Motive List (.motl/.em) generated in the previous step is loaded using the Place Object Chimera plugin^25^ to show position of subtomograms to be extracted.

In tomograms containing biological membranes, the signal of lipid bilayers is much stronger in the direction parallel to the tilt axis rather than the perpendicular, due to the missing wedge effect (Fig. 2a). Consequently, the membranes on the *XY* plane are well defined but the membranes along the *Z*-axis are often not visible or poorly defined (Fig. 2a). As part of our COPII workflow, we correct for resolution anisotropy with a software package called IsoNet which uses convolutional neural networks (CNNs) to restore information lost due to the missing wedge effect up to a resolution of ∼30 Å.^18^ Furthermore, IsoNet denoises tomograms, which increases their contrast and SNR using an adaptation of the noise2noise algorithm.^19^ The result is that biological membranes become clearly visible and well defined for all views (Fig. 2a), which in turn makes the process of picking membrane-associated particles more straightforward both for user-driven and automated picking strategies.

It should be noted that IsoNet-treated tomograms are useful for visualisation and particle picking, but they should not be used for high-resolution STA. In fact, the software is most effective on tomograms with a pixel size of at least 10 Å, so tomograms should be binned to that pixel size for IsoNet filtering and particle picking. Additionally, the parameters used in IsoNet do require some optimisation to yield the best possible results. Specifically, we recommend optimisation of the subtomogram extraction mask(s) and the SNRFallOff parameter to maximise contrast and minimise blur. A tutorial on using IsoNet can be found online (https://github.com/Heng-Z/IsoNet/blob/master/IsoNet%20tutorial.pdf) and another guide is included as part of the step-by-step guide in the ESI.†

### The optimal picking strategy for membrane-associated particles

In data suitable for STA, multiple copies of the particle of interest are present within the tomograms. The coordinates of each particle should be correctly and efficiently identified *via* effective particle picking strategies. To generate a high-resolution map, the particle picking strategy must be able to accurately find the specific complex of interest and be able to distinguish it from noise and particles of different nature. This process is made more challenging by the low SNR of the tomograms and the missing wedge. Furthermore, cryo-ET datasets can be very large, consisting of hundreds of tomograms, which can lead to the picking process taking on the order of weeks to months. Therefore, particle picking strategies must be optimised to be as high throughput as possible.

For membrane-associated proteins, the location of the lipid bilayer within tomograms can be used to assist in finding the particles of interest. This is highly effective due to several factors. Firstly, the high SNR of membranes simplifies the identification of regions in the tomogram where membrane-associated particles are located, reducing the probability of selecting false-positives. Secondly, membrane-associated particles typically adopt a consistent orientation relative to the membrane; this allows the approximation of at least two of the particle’s Euler angles, which speeds up and improves the accuracy of the alignment process.

### Picking particles: ordered, straight tubes

When COPII proteins are incubated with a non-hydrolysable GTP analogue and giant unilamellar vesicles (GUVs), coated membranes are generated. Many consist of coated tubes, which are long and straight.^7^ The COPII inner coat is closely associated with the membrane and forms a tightly packed and ordered lattice. Our picking strategy for these tubes utilises the Pick Particle plugin for Chimera^20^ to define the coordinates of each tube’s membrane within the tomograms (Fig. 2b–d). Here, the user traces points along the axis of the tube and defines the tube radius/diameter yielding a tube model (Fig. 2b and c). At this point, the exact coordinates of each COPII inner coat particle on the membrane are unknown. Therefore, we set the sampling rate in the Pick Particle plugin to define subtomogram coordinates at a sampling rate that is higher than the expected distance between neighbouring particles (Fig. 2d). Oversampling ensures that each COPII particle is contained in at least one subtomogram. The Pick Particle plugin also assigns orientations to subtomograms, normal to the membrane. The output is a table of subtomogram coordinates and orientations in Motive List (.em) format which can be used in the AV3 (ref. 21) subtomogram alignment and averaging package, or can be converted for use in different software such as Dynamo.^12^ A step-by-step guide to using the Pick Particle plugin is provided in the ESI.†

### Picking particles: irregularly shaped membranes

**Fig. 3:**
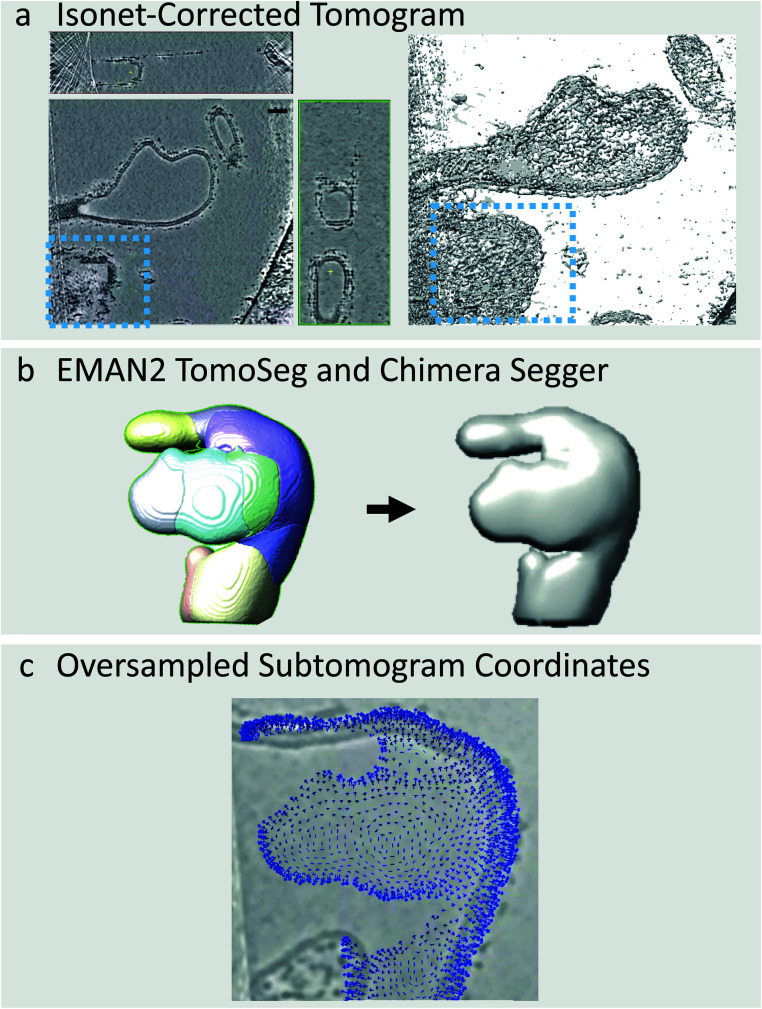
Picking particles from irregular membranes. (a) IsoNet-corrected tomograms are visualised in IMOD/3dmod (left). A surface rendering of the same tomogram can also be visualised in UCSF Chimera (right). This tomogram contains irregularly shaped coated membranes. (b) Individual objects are segmented using EMAN2 TomoSeg, and surfaces of each segmented membrane object are generated in Chimera. (c) These surfaces are used as a basis to pick oversampled coordinates on the membrane, and to assign initial orientations normal to it.

The COPII coat can also induce membranes to adopt morphologies other than long, straight tubes such as vesicles, and irregular shapes which do not match any conventional geometric pattern (Fig. 3a). Vesicles can be picked in a similar manner to tubes using the Pick Particle plugin and using the Sphere object style rather than the Tube object style, as carried out previously by Qu *et al.*^20^ However, it is not possible to use this plugin for irregularly-shaped membrane morphologies. One alternative option is to manually trace the membrane through each slice of the tomogram, as available in the Dynamo package,^22^ but this is prohibitively time-consuming for large datasets. To deal with this issue, we have developed a semi-automated workflow to pick particles on irregularly shaped membranes by combining IsoNet filtering, the CNN-driven automated tomogram segmentation tool provided in EMAN2,^23^ the Segger plugin in Chimera, and in-house scripts (provided in the ESI†).

EMAN2 contains a CNN-driven automated tomogram segmentation tool which can be used to identify the membranes within a tomogram. This tool requires that the user trains the neural network by clicking positions in the tomogram where membrane is present or absent. The CNN then finds membranes in all tomograms in the dataset automatically (Fig. 3). We find that this segmentation tool is far more effective on IsoNet-treated tomograms as the membrane is more prominent in all directions. An excellent guide to using the EMAN2 segmentation tool is provided by the authors (https://blake.bcm.edu/emanwiki/EMAN2/Programs/tomoseg).^23^

Once the tomograms have been segmented, we use the Chimera Plugin Segger to identify individual ‘objects’ (Fig. 3b). For example, if a tomogram contains two tubes and two irregularly shaped membrane buds, we select and group the membranes of each individual object together, followed by creation of individual densities using Segger’s ‘extract regions’ tool (Fig. 3b). Individual objects are then opened in Chimera and subjected to Gaussian filtering using the Volume Filter tool to smoothen the density of the membrane. This procedure is done manually on a tomogram-by-tomogram basis, so it is time consuming, however, it is a lot faster and less subjective than manually tracing each object. We then use in-house scripts, coupled with AV3 functions,^21,24^ (see https://github.com/EuanPyle/Membrane_Associated_Picking/) to define subtomogram coordinates over the membrane surface at a set sampling. These scripts assign orientations to each subtomogram normal to the membrane surface, and provide an option to assign directionality towards or away from the centre of mass of the whole membrane object (Fig. 3c). As before, the output is a table of particle coordinates and orientations in Motive List (.motl/.em) format. Unwanted particles or regions can be deleted using the Place Object plugin in Chimera.^25^ A step-by-step guide to this process is provided in the ESI.† We also note that alternative methods for picking on tubes, spheres and other geometries, are implemented in the Dynamo package.^22^ Particle picking at defined distances from segmented surfaces an also be carried out using the Membranorama package.^26^

### Particle cleaning: initial particle averaging

From the previous steps, we now have coordinates and initial orientations of subtomograms for each membrane of interest within our tomograms. However, as we have ‘randomly oversampled’ the membrane, there will be more subtomograms across the membranes than there are real particles. Furthermore, the real particles will not be at the centres of subtomogram boxes, but rather in random positions with respect to the membrane plane. Subtomograms should therefore be extracted in boxes larger than the known distance between neighbouring particles on the membrane to ensure that an entire particle can fit comfortably inside a subtomogram. Additionally, some subtomograms may not have any particle present at all. In order to find the subtomograms in which particles of interest are present, to ensure that only one subtomogram is extracted per particle, and that the particles are all centred consistently across the dataset, we carry out an initial coarse subtomogram alignment and averaging step immediately after particle picking.

**Fig. 4:**
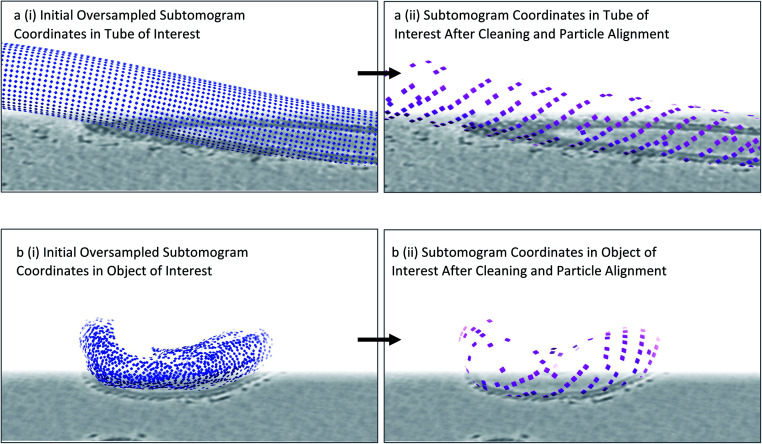
Identifying particle positions by subtomogram alignment. (a) (i) Oversampled coordinates are shown, equivalent to Fig. 2d. (ii) Upon alignment, neighbouring subtomograms that contain the same particle converge to a point which becomes the centre of a subtomogram. Duplicate subtomograms, as defined by subtomograms which are within a specified distance of one another, are deleted. (b) As above in (a), but for an irregularly shaped membrane. Note the appearance of patches of ordered lattice after this procedure is carried out.

We use the Dynamo package for initial subtomogram alignment and averaging,^12^ upon conversion of the output from previous steps from Motive Lists to a Dynamo table (using the dynamo__motl2table function in the Dynamo package). We extract the subtomograms, binned to a pixel size of ∼10 Å, as specified by the coordinates and orientations in the Dynamo tables using a particle extraction script (a template of which is provided in the ESI†). We then set up alignment projects for each individual object using a low-pass filtered structure of the COPII inner coat as starting reference. If a reference for the particle of interest is not available, a small number of particles can be aligned to generate one as described in the Dynamo guides^13^ (https://wiki.dynamo.biozentrum.unibas.ch/w/index.php/Main_Page). As the membrane is oversampled, multiple neighbouring subtomograms will converge to the same (or very similar) coordinates. The Dynamo option to set the ‘separation in tomogram’ to an appropriate distance (which should be less than the expected distance between two neighbouring particles) results in duplicate subtomograms (*i.e.* those that have aligned to similar coordinates) to be flagged for rejection from the particle pool. This reduces the number of subtomograms by minimising redundancy, and ensures subtomograms are centred similarly to the reference provided (Fig. 4). However, at this stage, there will still be a large number of subtomograms with either no real particle inside or a bad quality particle. As a result, we always carry out further particle cleaning steps.

### Particle cleaning: cross-correlation cleaning

**Fig. 5:**
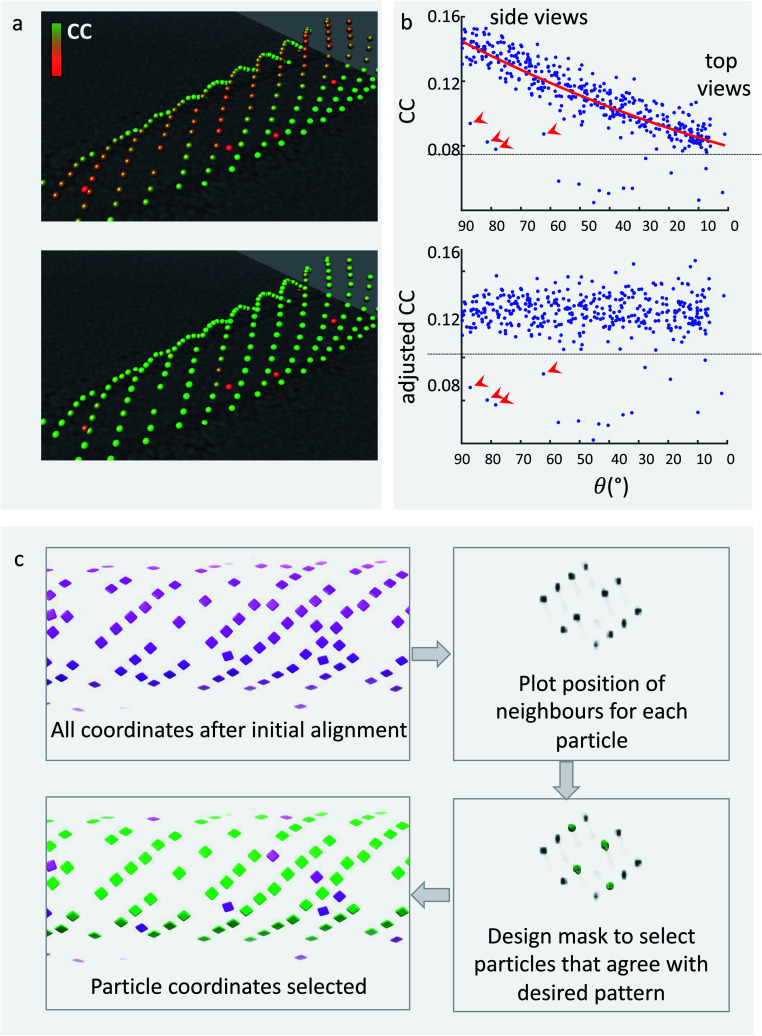
Cleaning the dataset to retain only ‘real’ particles. Top: (a) particles around tubular membranes have cross-correlation scores with a common reference that are dependent on their orientation: top views have lower scores (red) due to lower SNR of the membrane in that orientation, while side views have the best cross-correlation (green). (b) Plot of CC coefficient as a function of the tilt angle. A polynomial is fitted (red line). Red arrows indicate ‘bad particles’, that have clearly lower CC scores but still fall within a retention threshold designed to include side views (black line). Bottom: (a) after adjusting the CC score using the polynomial fitted in (b), real particles have uniform scores across tilt angles, while outliers (bad particles) retain a low CC score. (b) The plot of CC scores as function of tilt angles shows that a single-value threshold can now be used to eliminate bad particles (red arrows). (c) Particles arranged in a lattice can be cleaned using their arrangement. Top left: all particles around a COPII tubule after initial alignment. Top right: plot of the positions of neighbouring subtomograms, showing a preferential pattern. Bottom right: the plot is used to create a mask around expected lattice arrangement positions. This mask is used to select only particles that have at least one neighbour in one of the expected positions. Bottom left: the particles that are retained after this filtering (green) clearly arrange in a clean lattice.

Dynamo subtomogram alignment and averaging projects seek to maximise cross-correlation (CC) between subtomograms and reference(s) to find the optimal relative orientation of each subtomogram. The distribution of CC values across the aligned particle dataset is expected to reflect different degrees of similarity of each particle with the reference. A CC threshold can therefore be used to remove particles which are not similar to the reference, and are likely to be bad particles, proteins of different nature, or noise. However, setting the best CC threshold is not trivial for two main reasons. Firstly, the distribution of CC values for each membrane object differs due to variation in tomogram quality, ice thickness, defocus, and object orientation. Therefore, we optimise the CC threshold for individual membrane objects. Secondly, the membrane contributes heavily to the CC score of each subtomogram; however, due to the missing wedge effect its signal differs depending on orientation (Fig. 5a). As a result, subtomograms which have the same quality of particle and precision of alignment, but are in different orientations, can have different CC scores (Fig. 5a). This leads to bad particles sitting on membranes at the equator (side views) to have cross-correlation values often higher than real particles on top and bottom of the membrane. A single-value threshold would therefore either include bad particles or eliminate good top views and eventually induce resolution anisotropy in the subtomogram average. To counteract this effect, we have developed a method to re-weight the CC scores of subtomograms from each individual object to account for the missing wedge effects. The dependence of the CC value on the angle of latitude, commonly referred to as theta, is plotted and a polynomial curve is fitted (Fig. 5b). The fitted curve parameters are then used to adjust the CC values so that they are normalised across the whole range of theta angles. This allows to remove bad particles that have artificially high CC values by virtue of being ‘side views’ by application of a single-value threshold. The relevant script, alongside a guide on how to use it, is provided in the ESI.†

We use the Place Object plugin for Chimera^25^ to inspect, adjust, and save the CC-thresholds for each membrane object. We open the Motive List file for an object, and experiment with the lower and upper CC thresholds until the optimal balance between keeping good particles and removing bad particles can be found. Place Object saves a table of particle coordinates and orientations in Motive List format which we convert back to a Dynamo table.

### Particle cleaning: neighbour analysis

Many membrane-associated particles are organised into a regular pattern.^6,7,20,27,28^ In the case of COPII inner coat subunits, the arrangement into a pseudo-helical lattice means that each particle is surrounded by other inner coat particles at set distances and angles. Any subtomogram of the COPII inner coat which is not within a reasonable range from the expected position relative to neighbouring subunits is highly likely not to be a real particle and will have a detrimental effect on the subtomogram alignment and averaging process. We can exploit this information to clean the particles further.

We use a script which produces a plot of the most common positions of neighbouring subtomograms with respect to each aligned subtomogram, similar to the method used by Kovtun *et al.*^29^ If there is any ordered pattern, then this plot will show clear peaks of density when visualised in software such as UCSF Chimera. The plot can be filtered and binarized to obtain a mask that only contains the expected positions of neighbours. This can be used to select only particles that have at least one neighbour in one of the expected positions given the regular lattice, thereby removing from the dataset any lone or misaligned complexes (Fig. 5c).

A template for the neighbour analysis script is provided in the ESI† alongside a step-by-step guide on its use.

### Subtomogram alignment and averaging

Once the subtomograms have been picked and cleaned, they are now ready for subtomogram alignment and averaging. For this, we either use Dynamo^12^ or convert our Dynamo tables into .star files ready for import into RELION v4.0 (currently in Beta testing) using the dynamo2relion tool (https://github.com/EuanPyle/dynamo2relion). An extensive analysis of subtomogram alignment and averaging packages has been covered by recent reviews.^3,4^

## Discussion

The workflow provided (Fig. 1) is a suggested template for particle picking of tightly-packed membrane-associated complexes within a subtomogram averaging project. However, users may choose to seek alternative solutions to a number of these steps, which we discuss below.

Alternative options to IsoNet for increasing the SNR of tomograms have been successfully used in many studies. Tomograms can be filtered, for example using the SIRT-like (Simultaneous Iterative Reconstruction Technique) option in IMOD or Dynamo,^10–12^ or simply binned. Other methods that successfully denoise tomograms, boosting the SNR, use machine learning such as Topaz-Denoise, or Warp.^30,31^ However, none of these methods improve on the missing-wedge dependent anisotropy of the signal, which we found is fundamental in particular for picking from irregularly shaped membranes.

There are several alternative particle picking strategies to surface-based picking such as manual picking, template matching, and convolutional neural network (CNN)-guided picking. Manual picking involves the user visualising each tomogram in detail and selecting coordinates where the particle of interest is clearly identifiable. This process can be prohibitively time-consuming, especially if the number of particles in the tomograms is in the thousands or higher. This alone makes manual picking unattractive for most high-resolution STA workflows. Furthermore, accurate manual picking relies on the particle of interest being large and recognisable enough so that it can be clearly identified in low SNR tomograms. However, manual picking can be the best option if complexes of interest are sparse.^32,33^ Template matching is an automated particle picking strategy which uses a low-resolution template volume to search for similar particles within tomograms through cross-correlation. Template matching is considerably faster and less user reliant than manual picking. However, it has so far been demonstrated only for large complexes.^4^ CNN-guided picking integrates deep-learning into the particle picking process. CNNs are trained to recognise the particle of interest through the user identifying regions in a small number of tomograms with and without particles.^34^ After training, the CNN is used to find particles automatically across all tomograms. Recent advances in this area, such as DeepFinder, have demonstrated promising results for particles over 500 kDa.^34^ As the field of CNN-guided particle pickers improves, we expect that this method of particle picking will supersede other manual or semi-automated particle picking strategies due to both their improvements in accuracy and their relative speed.

We believe that CNN-guided picking holds the most promise for overcoming the most common issues with particle picking such as the time spent picking particles and distinguishing between different particle populations in noisy tomograms. Firstly, deep learning methods have been shown to display excellent pattern recognition capabilities, which effectively form the basis of particle identification.^34^ Secondly, CNN-guided picking has been shown to effectively distinguish between structurally similar yet separate particle populations.^34^ Finally, the semi-automated nature of CNN-guided picking effectively reduces the amount of time the user spends picking particles. As particle picking is often the step in the typical STA workflow which requires the most user input and expertise, improvements in automated and non-user biased picking strategies such as CNN-guided particle picking should be prioritised for the benefit of the STA field.

## Conflicts of interest

There are no conflicts to declare.

## Supplementary Material

FD-240-D2FD00022A-s001
