# Supplementary material for: Strategies for picking membrane-associated particles within subtomogram averaging workflows
Source: Faraday Discuss. 2022 Apr 12;240:101–13. doi: 10.1039/d2fd00022a (PMC9642003; doi:10.1039/d2fd00022a)
Supplement: FD-240-D2FD00022A-s001 [file FD-240-D2FD00022A-s001.pdf]

Electronic Supplementary Material (ESI) for Faraday Discussions.
This journal is © The Royal Society of Chemistry 2022

Particle Picking Step-By-Step Guide

Requirements

The following programs are required to follow this protocol:

MatLab (tested on R2019)

Dynamo (tested on v1.1.509)

IsoNet (tested on v0.1)

IMOD (tested on v4.9.0)

Chimera (tested on v1.15) with Pick Particle and Place Object plug ins

EMAN2 (tested on v2.3 and v2.91)

The scripts and sample files used in this protocol can be found in https://github.com/EuanPyle/Membrane_Associated_Picking.

Pre-Processing and Tomogram Generation

We pre-process our data and generate our tomograms using the following program, a guide on how to use it is included in the README. You can pre-process your data whichever way you see fit. When generating tomograms for particle picking, the tomograms must be binned to a pixel size of at least 10 Å to be suitable for input into IsoNet. We recommend using weighted back-projection for tomogram reconstruction. Keep the tomograms in a directory named ‘tomograms’. They should use the following naming convention: TS_[tomogram/tilt series number].mrc, e.g. TS_100.mrc.

IsoNet

Official IsoNet documentation can be found here. You should use tomograms of a pixel size of at least 10 Å as an input. The tomograms generated by IsoNet will be used for particle picking and initial subtomogram averaging, but they will not be used for high-resolution subtomogram averaging later in the workflow (i.e. for averaging subtomograms where the pixel size is less than 10 Å). Do not CTF correct or apply dose-weighting to your tomograms as IsoNet will carry out CTF deconvolution. We always generate a new IsoNet model for each dataset, regardless of whether the sample is similar or not. Here, we describe

additional tips and suggestions that we utilise when using IsoNet which will supplement the advice given in the official guide.

To easily edit the .star files generated (e.g. for entering the estimated defocus values of the 0° tilts in the tomogram .star file), we recommend either using a star file editor using Python and Pandas or using the ReadStarFile and WriteStarFile MatLab functions provided here.

Do not skip CTF deconvolution unless your data was acquired with a phase plate. To optimise CTF deconvolution, vary the --snrfalloff value between 1 and 0.25, and compare the results. You want to optimise SNR falloff to gain maximum contrast and minimise blur.

Before generating the mask, if you have many tomograms in your dataset, select ~5 tomograms with which you want to train the Isonet neural network on. You should pick these tomograms carefully so that they: are found across a range of defocus values, include all shapes/objects typically found in your sample, be good quality tomograms with not too much contamination. Try to avoid tomograms with visible carbon edges as this can interfere with the masking procedure later. Set the value for _rlnNumberSubtomo in the tomogram .star file to 0 for each tomogram other than the tomograms you wish to train on. The _rlnNumberSubtomo for the tomograms you wish to train on should total ~300 subtomograms (i.e. if you wish to train the neural network on 3 tomograms, the rlnNumberSubtomo should be ~100 for each).

We want the mask to mostly include the membrane regions of our tomograms. When generating the mask, we recommend using the flag '--z_crop' to avoid masking the top and bottom of the tomograms in the Z axis as these regions are often empty and will yield no useful training data. We generally use a value of 0.2 to crop 20% of the tomogram. Optimise the masks by changing --density_percentage and --std_percentage values until the mask selects at least the vast majority of the membranes in your tomogram. Some noise will inevitably be included in the mask: we found that the inclusion of noise did not have a detrimental impact of the quality of IsoNet training provided the majority of the mask was focused on the membrane.

When extracting subtomograms, use the flag '--tomo_idx' to select the indices of the tomograms you wish to train IsoNet on. We did not find that adjusting _rlnCropSize or _rlnCubeSize from the default values gave improved results.

Keep the generated tomograms in a directory named 'tomograms'. They should use the following naming convention: TS_[tomogram/tilt series number].mrc, e.g. TS_100.mrc.

Particle Picking: Defining Initial Subtomogram Coordinates

We are now going to use the tomograms generated by IsoNet to define the initial coordinates of the subtomograms for particle picking. If you did not use IsoNet to generate tomograms, we recommend generating tomograms with a pixel size of at least 10 Å and using a SIRT (or SIRT-like) filter to increase the SNR and contrast to assist in picking.

We include two particle picking strategies here. The first uses the Pick Particle and Place Object Chimera plug ins, which can be downloaded from here. This should be used if membranes with the associated particle of interest are either regular vesicles or tubes. Tubes which are bent can be incorporated into this protocol, but tubes with substantial radius variations may be easier to pick using the second particle picking strategy for irregular-shaped membranes.

Pick Particle Strategy

Open an IsoNet-treated tomogram in Chimera. In the Volume Viewer toolbar, navigate to Features/Coordinates. Enter the pixel size in the Voxel size box. In the Volume Viewer toolbar, navigate to Features/Planes. Under the Style option, select solid. Directly under the Plane option, select One. Adjust the threshold sliders to properly adjust the grey levels in the tomogram. Use the plane slider to navigate through the Z axis of the tomogram.

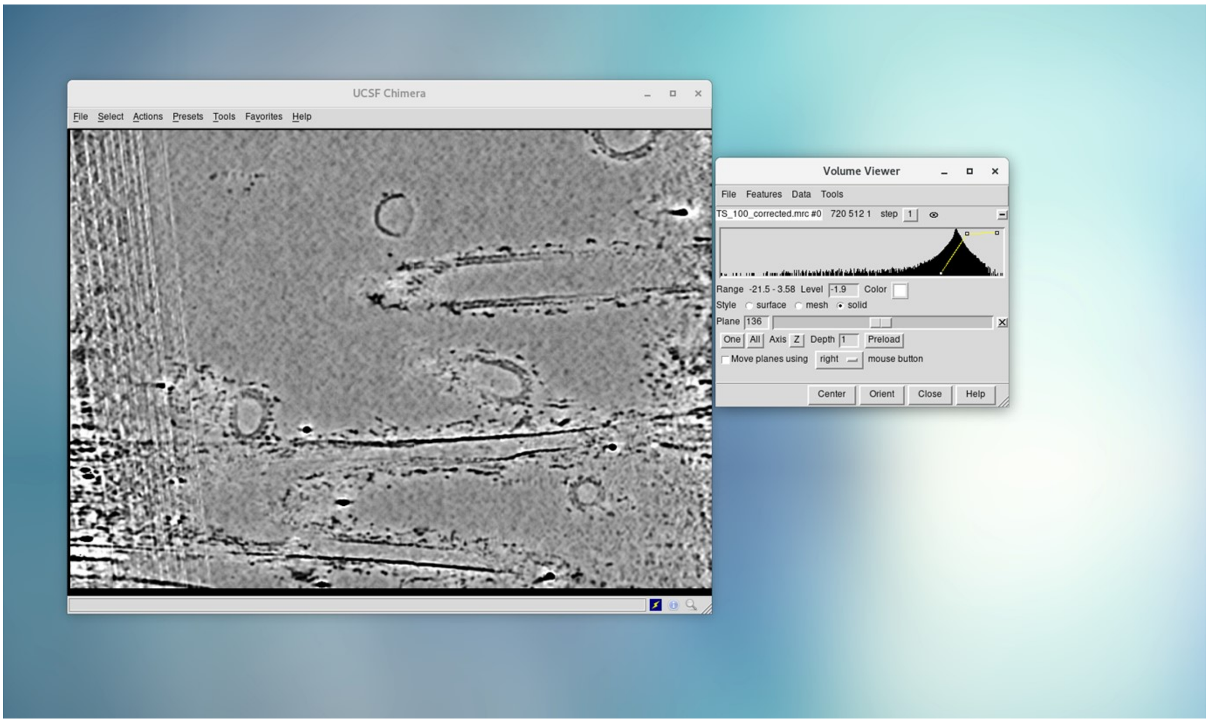

In the Volume Viewer toolbar, navigate to Tools/Volume Tracer. In the Volume Tracer toolbar, navigate to Mouse, and make sure only 'Place markers on data planes' is selected. Tick 'Place marker using [Your choice!] mouse button'. Start a new marker set by navigating to File/New marker set.

For tubes, adjust the plane in the volume viewer to ascertain the Z-height of the middle of the tube. Using the mouse button you selected earlier, place markers along the tube axis, from one end of the tube to the other, adjusting the Z-height as necessary to ensure the markers are always placed on the tube axis. One can also adjust the radius of the marker to 5-50 to make sure the points are clearly visible. You can delete markers in Actions/Delete Markers. Bent tubes can be traced as well as straight tubes. When you have finished placing markers for one tube, in Volume Tracer toolbar, navigate to File/Save marker set as, and save the tube. Name it under the following convention: TS_[Tomogram_Number]_object_[Object_Number], e.g. TS_100_object_1 for the first tube in tomogram 100, and TS_100_object_2 for the second tube in tomogram 100. This will write a TS_X_object_X.cmm file in the tomograms folder. In Volume Tracer, close the marker set by navigating to File/Close marker set. For the remaining tubes in the tomogram,

start a new marker set by navigating to File/New marker set, and pick the next tube in the tomogram.

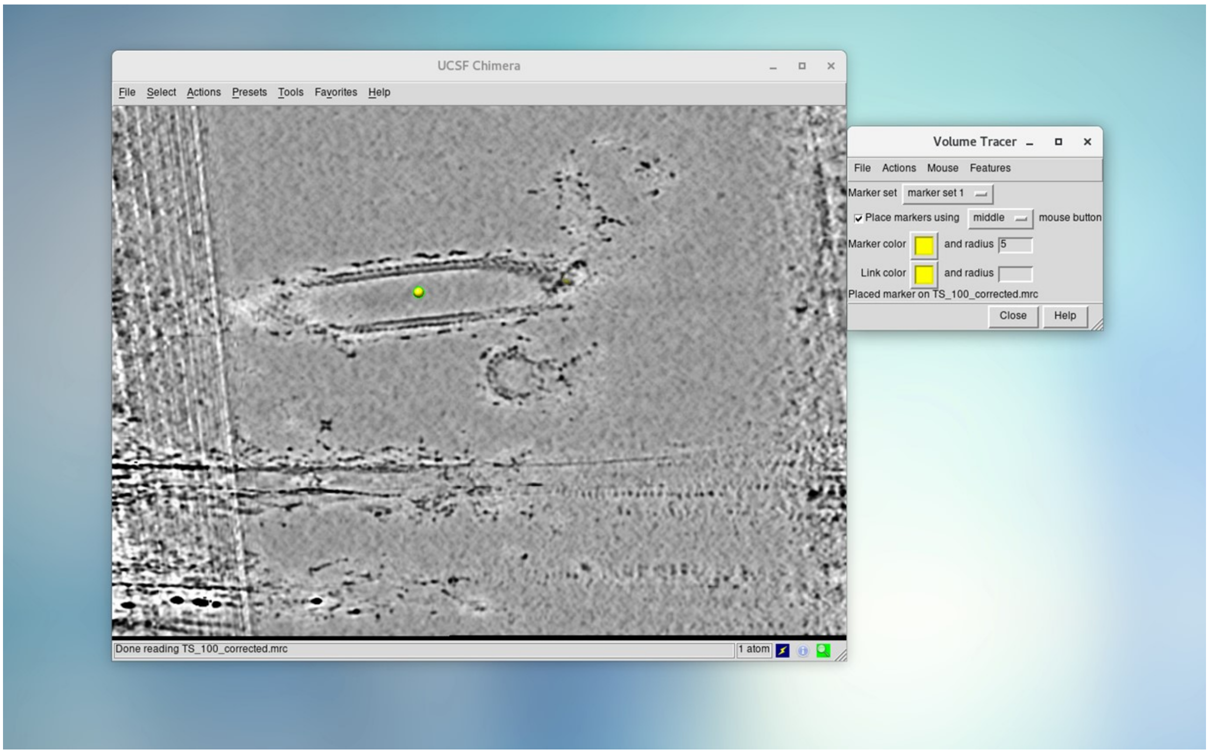

Navigate to Tools/Utilities/Pick Particle in the main Chimera toolbar. In the Pick Particle window that appears, insert the tomogram number (e.g. TS_100 would be 100) in the Tomogram ID box. Browse for one of the marker files (e.g. TS_100_object_1.cmm) under Marker File, tick on 'Tube' for Object Style, and click 'Display'. You will see the previously picked tube axis appear as a yellow line. Adjust the radius with the slider so that the line expands to place the yellow edge of the tube where you wish the centres of picked subtomograms to be. Use the volume viewer to go up and down in Z to check the radius is optimised. Adjust sampling to required oversampling rate (i.e. the number of pixels between which subtomograms are extracted on the tube surface) in tangential and axial sampling. Write this number in **unbinned pixels** (if your tomogram is binned by 8, you will have to multiply the number of pixels in your tomogram by 8). The oversampling rate should be less than the known distance between two particles on the membrane surface. Click 'Save' which will automatically produce a Motive List (.em) file containing particle coordinates (e.g. TS_100_object_1_tube.em). Ignore TS_100_object_1.em, this is the marker set.

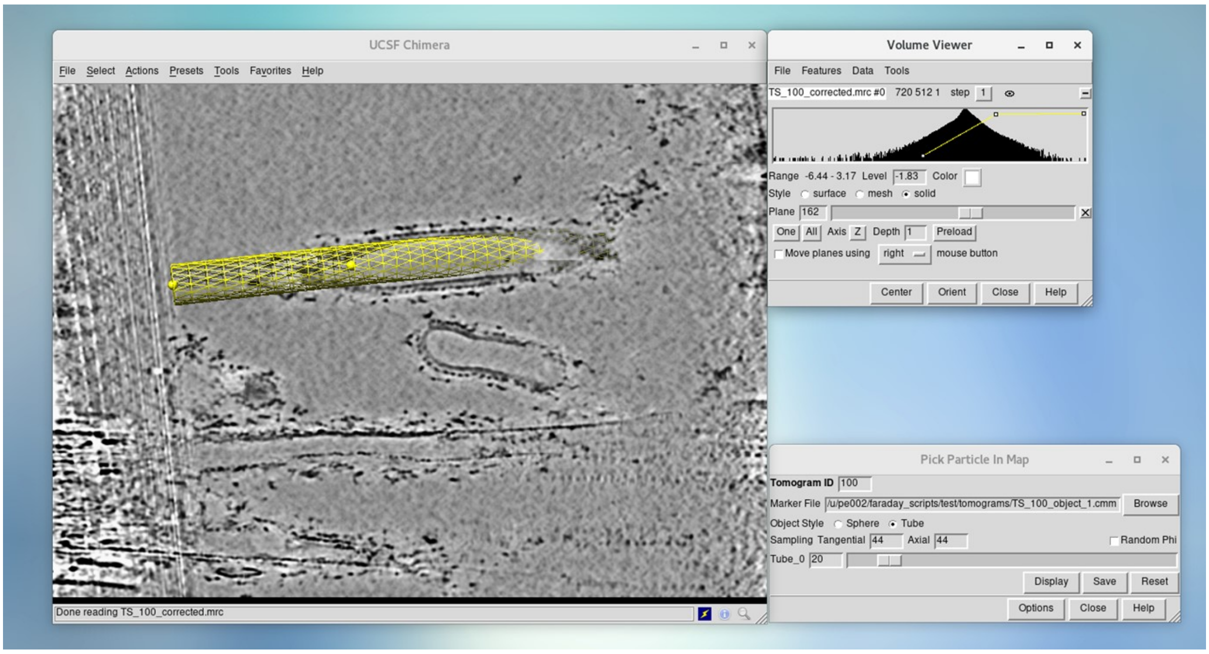

To check subtomograms coordinates are spaced as you intend, open the Place Object plug in via navigating to Tools/Utilities/Place Object. In coordinate file, open the Motive List file (e.g. TS_100_object_1_tube.em), and then 'Apply'.

Generate objects for all tubes/spheres in the tomogram. Close the session and repeat this process for all tomograms.

For vesicles, repeat the above, but only use one marker for each vesicle. Place the marker in the middle of the vesicle. Use Sphere in Object Style.

Pick Particle has saved the oversampled coordinates (e.g. TS_100_object_1_tube.em) of your initial subtomograms as a Motive List which can be used in the AV3 subtomogram averaging package. We carry out the initial subtomogram alignment and averaging step in Dynamo and will convert the Motive Lists to Dynamo tables later.

When you have defined the initial coordinates of your subtomograms for your objects of interest in each tomogram, go to Averaging section of this protocol.

Irregular-Shaped Membranes Strategy

Before we can define the coordinates of our initial subtomograms on the membrane, we need to define the position of the membrane within our tomograms. We use the automated tomogram segmentation tool in EMAN2 to do this. The official guide to segmentation in EMAN2 is excellent, and can be followed step-by-step from here: https://blake.bcm.edu/emanwiki/EMAN2/Programs/tomoseg. Use IsoNet-treated tomograms as input, both in the training and for segmentation. Select the membrane, associated with your particle of interest, as the feature of interest. After the Apply to Tomograms step, check the segmentation overlaps correctly with your input tomograms by opening the resulting segmentation and the input tomogram in a Chimera session. The resulting segmented tomograms can be opened in Chimera. The membrane regions of interest should consist of strong density. Some noise will also be included, but this is not an issue unless it obscures the membrane regions of interest.

Now the tomograms are segmented to highlight the membranes, we can use this segmentation information to define the coordinates of our initial subtomograms. For each tomogram, carry out this loop of instructions to define subtomogram coordinates for each object of interest:

1. Open the Isonet-treated tomogram of interest using 3dmod and make a note of the individual objects which you wish to pick particles on
2. Open the EMAN2-segmented tomogram in Chimera and in the Volume Viewer toolbar, select Tools/Volume filter and apply a Gaussian filter to smoothen the density. We used a width value of 6. Ideally, the resulting density should be smooth over the membrane regions

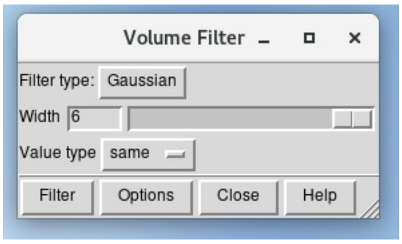

3. Open segger by navigating on the Volume Viewer toolbar to Tools/Segger. For the Map option, select the Gaussian filtered volume. At the bottom of the window, click options, select group by smoothing. Click Segment. This will split each membrane object into segments of different colours.

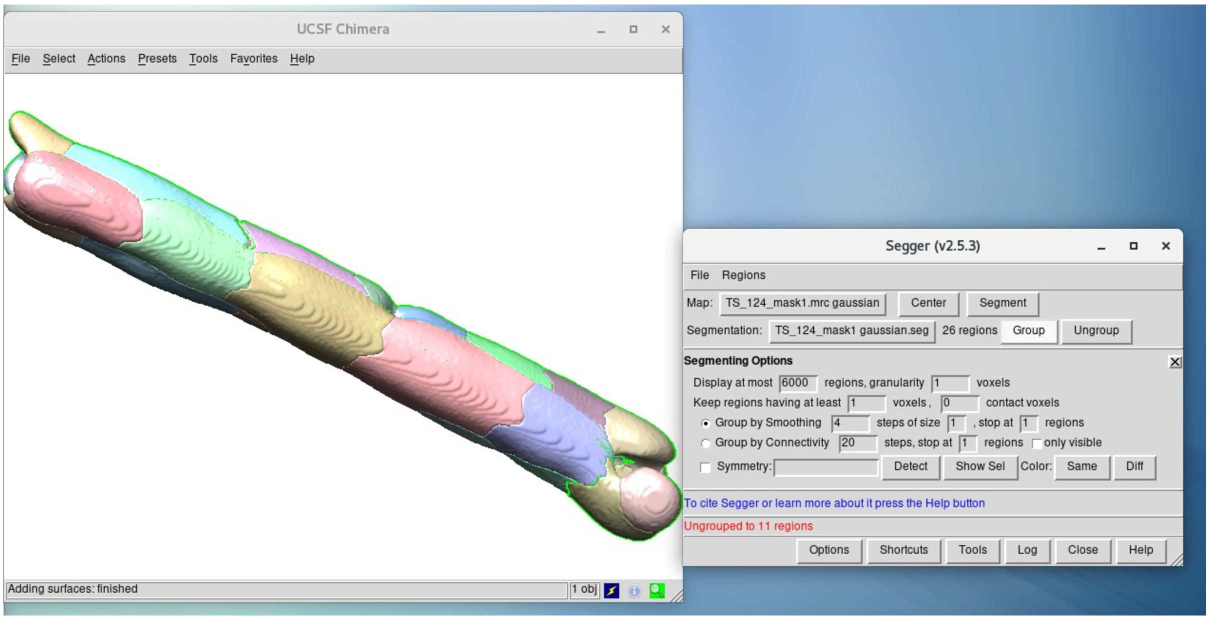

4. You now need to manually group each object/surface of interest individually, so each object is made from panels of the same colour. Hold Ctrl and Left click on one of the patches on the object of interest. Then Ctrl+Shift+Left click on each other patch in the same object. Turn all the panels the same colour by clicking group in the segger window. Repeat for each individual object of interest.

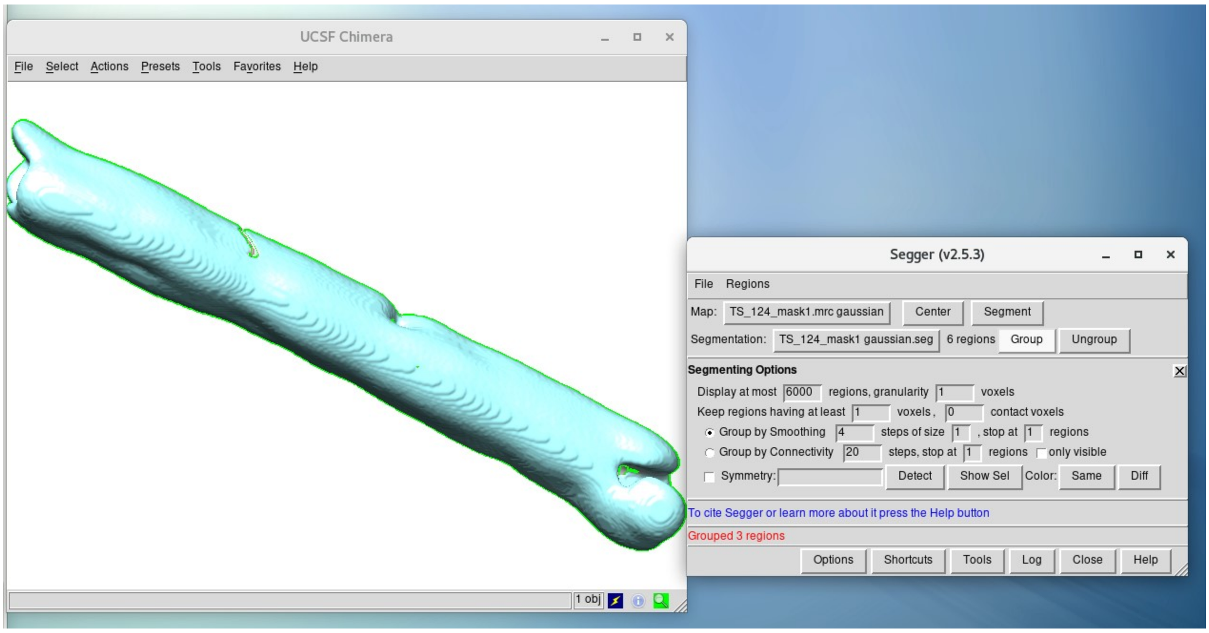

5. Once each individual object is grouped, in the segger window, click tools, and then extract. Under 'Extract densities from map' select the non-Gaussian filtered map. Under 'Dimensions of new map' select 'Same as map from which densities are extracted'. Highlight an individual object grouped by segger. Tick 'Save Map with name' and give an appropriate name to the extracted volume e.g.

TS_100_object_1.mrc. Click extract. Highlight the next object of interest, give it a different name, e.g. TS_100_object_2.mrc, and extract. Repeat for all objects of interest.

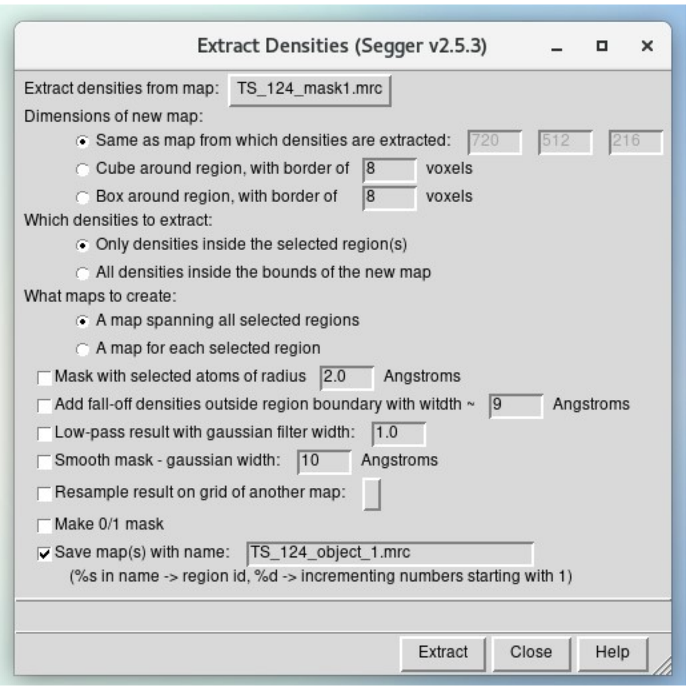

6. Open each extracted object in Chimera and carry out a Gaussian Volume filtering as before. Overwrite the saved volume file by navigating to File/Save Map As in the Volume Viewer toolbar. Repeat for each object. This saves a smoothed volume of the extracted object.
7. We now need to use these volumes as surfaces to define subtomogram coordinates on. The remaining steps of this loop will have to be run for each object in the tomogram of interest. Move the [create_gridpoint.m script] into the working directory. Open the script and edit it according to instructions in the script. Open MatLab, load Dynamo. Run create_gridpoint.m script in the MatLab terminal: create_gridpoint('TS_100_object_1',1);
where the first argument is a string containing the file name of the object you wish to define subtomogram coordinates on (with no .mrc extention), and 1 is the threshold (the ideal threshold will vary for each object, this will be optimised later. In the first instance, for each object, run this script using a threshold of 1).
8. The script takes a few minutes to run. Once finished, a Motive List (called TS_X_object_X.em) will be generated. In a Chimera session in which the tomogram of interest is open, the subtomogram coordinates should be visualised using the

Place Object plugin in Chimera. To open this plug in, in the toolbar of the Chimera window, navigate to Tools/Utilities/Place Object. Enter the Motive List for your object of interest as the coordinate file. Select ArrowZ as the Object Style and set the Voxel Size as 0.2. Click Apply. The coordinates where the script has defined that a subtomogram will be extracted should be superimposed on your tomogram as arrows. The direction of the arrow shows the orientation that each subtomogram will be assigned relative to the membrane. Next to the Visualisation option, click Class Number and adjust the class number between 1 and 2 with the slider below: this will give you the option of having your particles orientated pointing away from the membrane or towards the membrane. If the arrows are too small or large, adjust the voxel size. Check that the arrows superimpose well with the membrane of your object of interest. If the arrows do not superimpose well with the membrane, re-run the create_gridpoint.m script with a different threshold and try again.

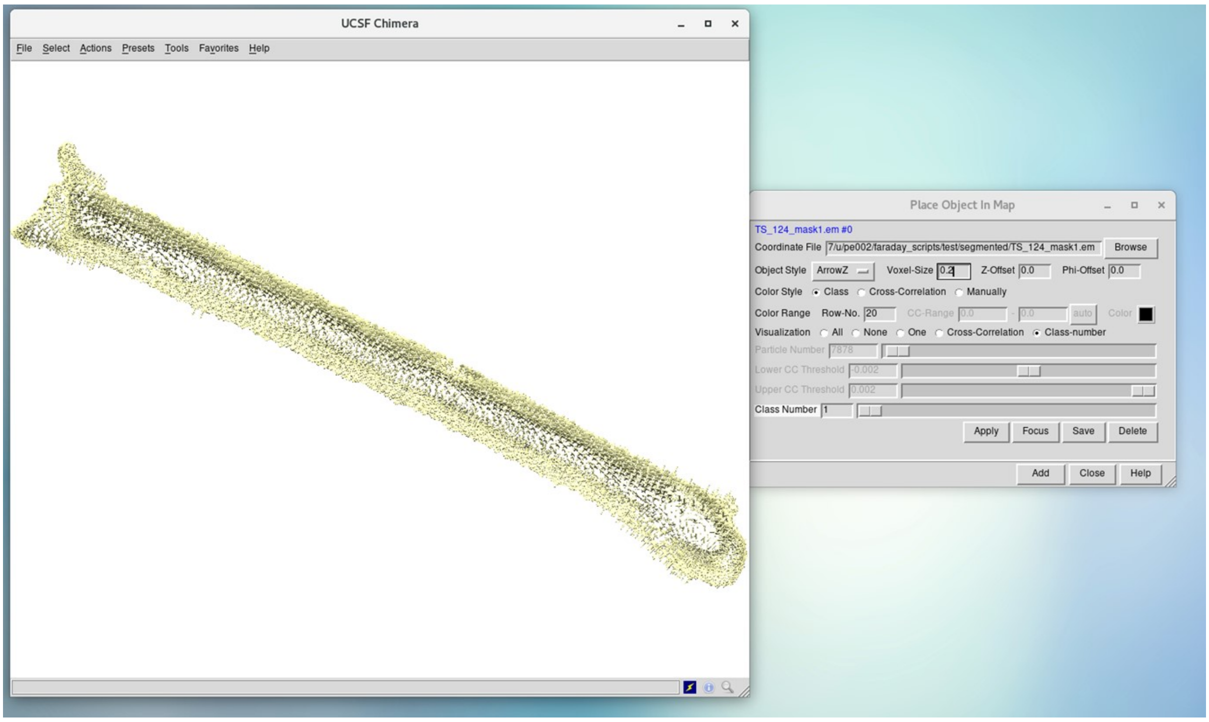

9. Once the arrows superimpose well on the membrane, hide the tomogram so you can clearly see the arrows denoting subtomograms. Identify clearly bad subtomograms, for example, arrows which are not close to the membrane or have the wrong orientation. You can delete bad subtomograms by holding Ctrl and left clicking on them, then in the Chimera command line entering: ~modeldisp sel. Once bad subtomograms have been deleted, click Save. This will save the coordinates of the

arrows as a Motive List under [File_Name]_display.em. Repeat this process for each object of interest in the tomogram.

Averaging

As the first step in particle cleaning (i.e. removing subtomograms with no particles, duplicates, or sub-optimal particles), we now carry out initial subtomogram alignment and averaging using Dynamo. The previous steps have generated Motive Lists for each object of interest of each tomogram. Move or copy all of these files into the same directory called 'initial_alignment'. This can be done with a command like: cp TS_*_object_*_tube.em ../initial_alignment; Each file should be named in the following naming convention: TS_[Tomogram_Number]_object_[object_number].em, e.g. TS_100_object_1.em. If you have carried out the Pick Particle Plug In protocol, you will have to remove the _tube or _sphere suffix from these file names. This can be done manually or by a command like this in bash: for f in ./*_tube.em; do newname=$(echo $f | sed -r 's/_tube//'); mv $f $newname; done;

We now need to convert these files from Motive Lists into Dynamo tables. Edit the motl2dynamo.m script according to the instructions inside the script. Open MatLab, load Dynamo (i.e. run dynamo_activate), and run the script in the MatLab terminal to generate the Dynamo tables of each object. Check the coordinates in the Dynamo table (columns 24 to 26) match the picked coordinates of your particles in the tomogram in 3dmod.

We now need to extract the subtomograms of each object from the tomograms. Copy extraction.m into the directory containing all the .tbl files for each object. Edit the extraction.m script according to instructions in the script. Again, in MatLab with Dynamo, run the script in the MatLab terminal. The particles for each object will be saved in separate directories. The orientations and coordinates of each particle are saved in crop.tbl which is found within the directory of each object.

We now need to carry out subtomogram alignment, where each subtomogram is aligned to a reference. Carry this out in the same directory you ran extraction.m in. If you do not have

a reference available, generate one in Dynamo by following this guide for your own data (do not use the training data set).

Open your reference in Chimera and check whether it is visible in the positive or negative threshold. To visualise your particle in the negative threshold, go into the Chimera toolbar and navigate to Volume Viewer/Features/Surface. For Cap at High Values, untick, and lower threshold into negative numbers. Our reference was in the negative threshold for Chimera. Double check you reference and particles are in the same threshold by opening a few extracted subtomograms in Chimera and repeating the threshold check. If your reference is in the opposite threshold to your particles, you can flip the threshold by typing into the Chimera command line: vop #0 scale factor -1; and save the resulting particle as the threshold flipped new reference.

We now need to generate alignment projects for each object. Open MatLab, load Dynamo, type in the Matlab terminal: dcp; to load the alignment project manager. Name this project initial_align. Click create new project when prompted. All future projects in this folder will use this project as a template. Open the particles window, select browse, open any directory where particles have been extracted, click open, wait until the directory name appears (this can take some time), then click ok. Open the table window, click browse file, go to the same directory, and select the crop.tbl file. Click Ok. In the template window, click browse, select your reference file, click ok. In the masks window, to the right of 'Create Ellipsoid' under alignment mask, enter the half the box size of your subtomograms, minus 1, three times. For example, for a box size of 32, enter: 15 15 15. Click create ellipsoid. Click copy alignment mask and fill with ones on the other options, then ok. Open numerical parameters. Enter settings as you see fit, either using our settings as displayed below or using the Dynamo tutorial to guide you.

	round 1	round 2	round 3
iterations	1	0	0
references	1	1	1
cone aperture	20	360	360
cone sampling	10	45	45
azymuth rotation range	20	360	360
azymuth rotation sampling	10	45	45
refine	4	5	5
refine factor	2	2	2
high pass	2	2	2
low	8	32	32
symmetry	c1	c1	c1
Particle dimensions.	32	0	0
shift limits	15 1...	4 4 4	4 4 4
shift limiting way	1	0	0
separation in tomogram	4	0	0
basic MRA	0	0	0
threshold parameter	0.20	0.20	0.20
treshold modus	0	0	0

Depending on your sample, you may want to increase the azimuth rotation range to 360 is you are unsure of the in-plane rotation of your particle relative to the membrane. The shift limits numbers should be the same as the numbers you entered during the 'Create Ellipsoid' step of mask generation. Setting the 'separation in tomogram' to the minimum number of pixels you would expect your particles to be separated by is a good way of deleting duplicate subtomograms which have converged to the same or similar coordinates. This is essential if your particles are oversampled. If you are unsure of this value, set a reasonable distance within which you would not expect to find more than one particles (i.e. the size of the particle itself in pixels).

Under computing environment, the correct settings will depend on your computational set up. Therefore, we cannot advise users on how to set this up. See the Dynamo wiki for help. We strongly advise using GPU acceleration.

Click check and unfold but do not click run. Test running this job using your computational setup to check for errors.

Now we need to generate an alignment project for each object using initial_align as a template. Copy make_projects.m into the working directory, this should be the same directory you ran extraction.m in. Open the script, edit it as per the instructions, and run it in the MatLab terminal with Dynamo activated. Then run each alignment job generated. Each job will generate an average of your aligned particles within that directory in, for example:
initial_align_TS_?_object_?/results/ite001/averages/average_ref_001_ite_0001.em.
Running this on a per-object basis will allow us to identify and clean objects containing ‘good’ and ‘bad’ particles later.

For each object, view the averages in Chimera. If your reference was in the negative threshold in Chimera, visualise it by going into the toolbar and selecting Volume Viewer/Features/Surface. For Cap at High Values, untick, and lower threshold into negative numbers to visualise your particles. Check the individual averages of the objects to ensure the alignments are working properly.

Particle Cleaning: CC-based Cleaning

We now want to remove subtomograms which contain noise or bad quality particles. We can do this initially by removing subtomograms which have a low cross-correlation (CC) to the reference. The optimal CC threshold below which we will delete subtomograms will vary from object to object, so the threshold must be set for each object individually. To set this threshold, we will need to visualise the CC of each subtomogram of each object. As we use Place Object for visualising the CC of each subtomogram, we will need to convert the Dynamo tables generated by the alignment of each object into Motive Lists. We also need to reweight the CC of each subtomogram depending on subtomogram orientation, as described in the main text. We can do both of these things by moving run_cc_weighting.m into the working directory, this should be the same directory you ran extraction.m in, and editing according to the instructions in the script, and running it in a MatLab terminal with Dynamo initialised. This will generate a CC-weighted Motive List in the same directory that the Dynamo tables from the alignment of each object are found (e.g. initial_align_TS_X_object_X/results/ite_000X/averages/cc_weighted.em).

For each object, we need to inspect the CC of the subtomograms and apply an ideal threshold using the Place Object plug in. Open Chimera and in the toolbar of the Chimera window navigate to Tools/Utilities/Place Object. Enter the Motive List for your object of interest (cc_weighted.em) as the coordinate file. Select ArrowZ as the Object Style and set the Voxel Size as 0.2. Click Apply. The coordinates and orientations of your subtomograms will be displayed as arrows. Next to the Visualisation option, click Cross-Correlation and Low CC threshold with the slider below: changing the value of the slider will remove subtomograms based on their CC to the reference. Find a threshold where the majority of the subtomograms are ‘good’, i.e. they superimpose of the membrane well and have orientations that make sense depending on the membrane. Do not be overly concerned by the presence of some ‘bad’ particles: these can be deleted at a later point. Once you have determined the best threshold for your object, note down the ideal threshold in cc_threshold.csv.

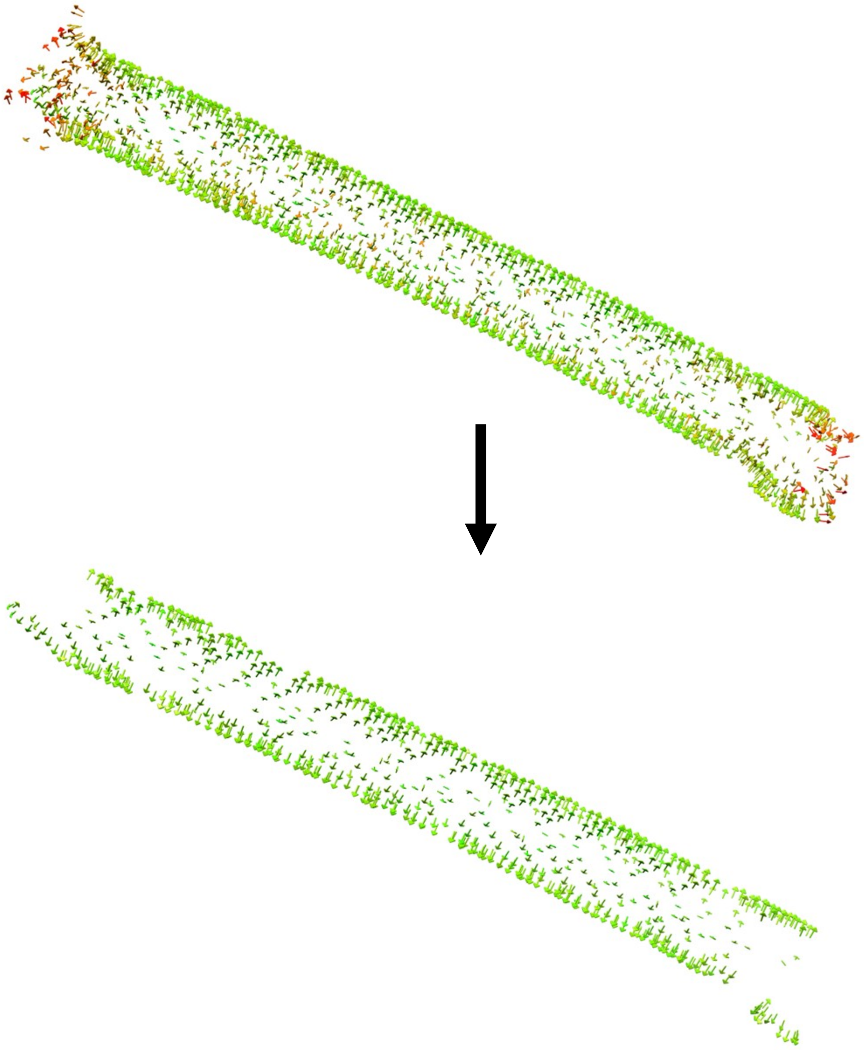

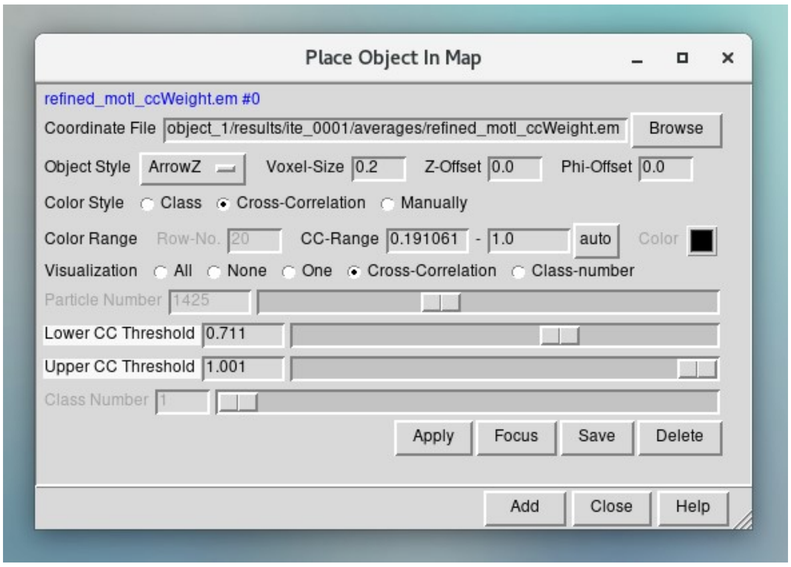
Place Object In Map
refined_motl_ccWeight.em #0
Coordinate File
object_1/results/ite_0001/averages/refined_motl_ccWeight.em
Browse
Object Style
ArrowZ
Voxel-Size
0.2
Z-Offset
0.0
Phi-Offset
0.0
Color Style
Class
Cross-Correlation
Manually
Color Range
Row-No.
20
CC-Range
0.191061
-
1.0
auto
Color
Visualization
All
None
One
Cross-Correlation
Class-number
Particle Number
1425
Lower CC Threshold
0.711
Upper CC Threshold
1.001
Class Number
1
Apply
Focus
Save
Delete
Add
Close
Help

In this document, each line contains information on a different object. The first value of each line denotes the tomogram (or TS) number, the second denotes the object number, and the third denotes the threshold. For example, if your data had two tomograms (TS_100 and TS_102), with one object of interest inside each, with an optimal threshold of 0.6 and 0.7, respectively, cc_threshold.csv should look like this:

100,1,0.6
102,1,0.7

Note the ideal CC threshold for each object in cc_threshold.csv. Now we can clean bad subtomograms from these objects based on their CC with the reference. Open and edit apply_cc_clean.m and follow the instructions in the script. Run the script in the MatLab terminal with Dynamo loaded. This writes a table of clean particles to each object directory.

Particle Cleaning: Neighbour Analysis

If your particles of interest are arranged in a regular, repeating lattice, it is possible to clean the particles further by carrying out Neighbour Analysis. Here, we establish the pattern of the lattice in the coordinates of our subtomograms, and delete particles which do not conform to this lattice.

To carry out Neighbour Analysis, copy run_neighbourplot.m into the working directory. Edit the script according to the instructions contained within. Run run_neighbourplot in a MatLab terminal with Dynamo activated. The output will be named 'all_neighbour.em' in the initial_alignments directory and will consist of a density map representing the most popular positions and distances of the subtomograms neighbouring each subtomogram. Open this file in Chimera. Adjust the threshold until the pattern is clear. If there is no pattern, then Neighbour Analysis will not be useful.

We now need to make a mask around the densities where we want to keep particles. See the picture below where you can see that subtomograms are a consistent distance away from one another.

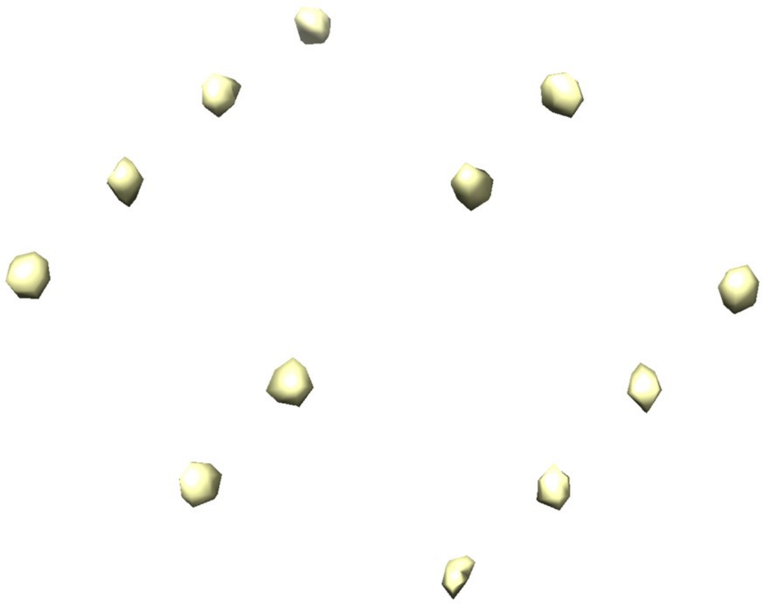

We want to create a mask around each of these blobs of density. We can do this using Segger. Open segger by navigating on the Volume Viewer toolbar to Tools/Segger. At the bottom of the window, click options, select group by connectivity. Click Segment. This will split each blob into segments of different colours. You now need to manually group each blob individually, if they are not so already, so they are all the same colour. Hold Ctrl and Left click on one of the blobs. Then Ctrl+Shift+Left click on each other blob in the pattern you wish to keep. Turn all the panels the same colour by clicking group in the segger window.

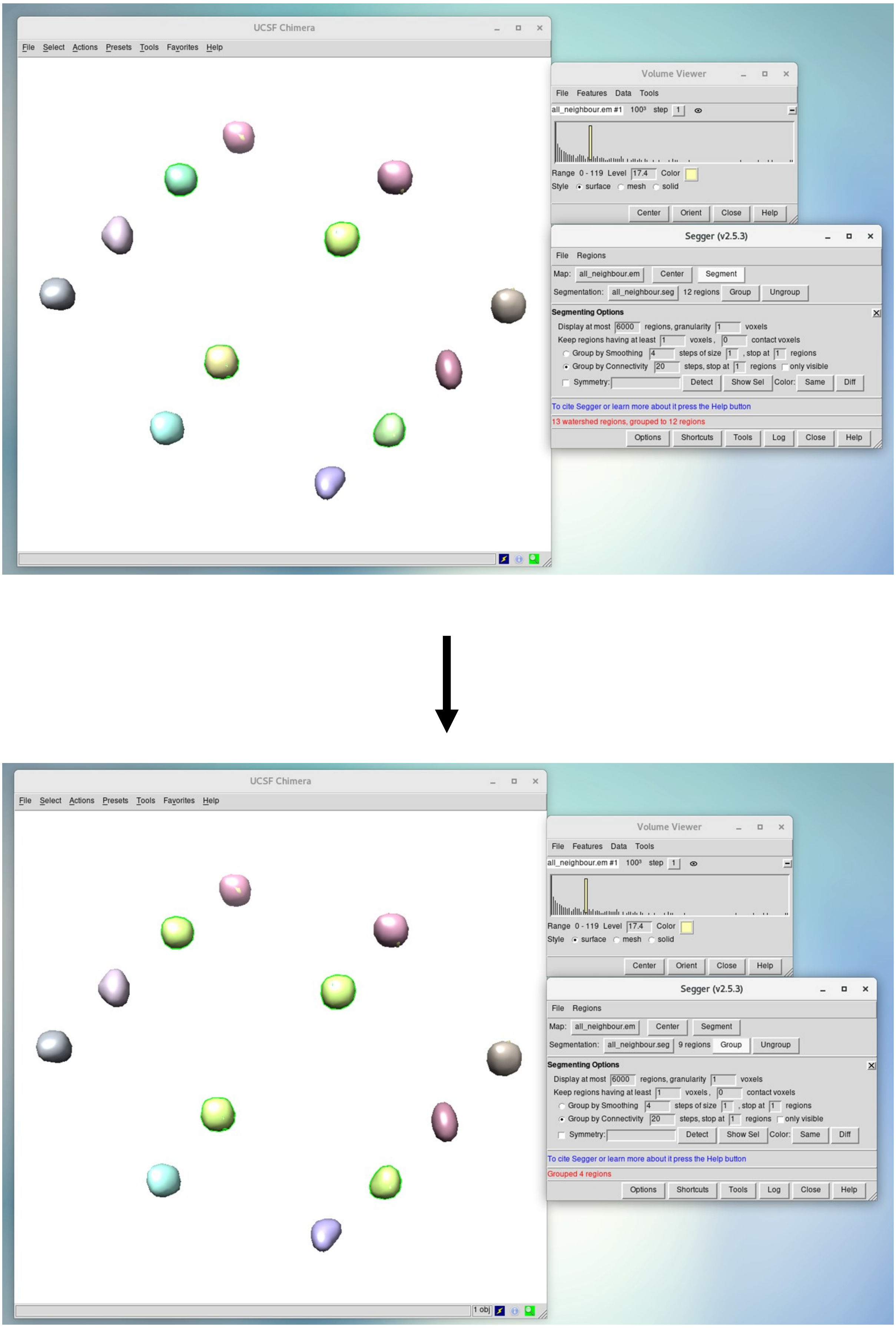
UCSF Chimera
File Select Actions Presets Tools Favorites Help
Volume Viewer
File Features Data Tools
all_neighbour.em #1 100³ step 1
Range 0 - 119 Level 17.4 Color
Style surface mesh solid
Center Orient Close Help
Segger (v2.5.3)
File Regions
Map: all_neighbour.em Center Segment
Segmentation: all_neighbour.seg 12 regions Group Ungroup
Segmenting Options
Display at most 6000 regions, granularity 1 voxels
Keep regions having at least 1 voxels, 0 contact voxels
Group by Smoothing 4 steps of size 1 , stop at 1 regions
Group by Connectivity 20 steps, stop at 1 regions only visible
Symmetry: Detect Show Sel Color: Same Diff
To cite Segger or learn more about it press the Help button
13 watershed regions, grouped to 12 regions
Options Shortcuts Tools Log Close Help
UCSF Chimera
File Select Actions Presets Tools Favorites Help
1 obj
Volume Viewer
File Features Data Tools
all_neighbour.em #1 100³ step 1
Range 0 - 119 Level 17.4 Color
Style surface mesh solid
Center Orient Close Help
Segger (v2.5.3)
File Regions
Map: all_neighbour.em Center Segment
Segmentation: all_neighbour.seg 9 regions Group Ungroup
Segmenting Options
Display at most 6000 regions, granularity 1 voxels
Keep regions having at least 1 voxels, 0 contact voxels
Group by Smoothing 4 steps of size 1 , stop at 1 regions
Group by Connectivity 20 steps, stop at 1 regions only visible
Symmetry: Detect Show Sel Color: Same Diff
To cite Segger or learn more about it press the Help button
Grouped 4 regions
Options Shortcuts Tools Log Close Help

In the segger window, click tools, and then extract (sometimes this is under Regions/Extract Densities). Under 'Dimensions of new map' select 'Same as map from which densities are extracted'. Tick 'Save Map with name' and name the file all_neighbour_pre_bin.mrc. Click extract. We now need to binarise this density. Open all_neighbour_pre_bin.mrc in Chimera, find a threshold where the pattern is clear. Make a note of this threshold. Open Matlab with Dynamo loaded. Type: bin=dread('all_neighbour_pre_bin.mrc') to load the mask. Binarise the mask by typing: bin=dynamo_binarize(bin,[threshold you optimised]); e.g. bin=dynamo_binarize(bin,7); then write the mask by typing: dwrite(bin,'binarised_mask.mrc'); and exit Matlab.

We now edit and run run_neighbour_cleaning.m using the mask just generated as an input. This will produce a table (neighbour_cleaned.tbl) for all objects where all points that were not within the mask boundary are eliminated. We can now use these tables as cleaned particle coordinates for further subtomogram alignment and averaging to high-resolution.

Further Processing to High-Resolution

Make a single table containing all particles using make_full_table.m. Edit the script according to the instructions in the script and run in a MatLab terminal with Dynamo loaded.

Once you have a single Dynamo table containing all your good quality particles, you can either continue to process your data using Dynamo on lower pixel size tomograms to get your structure to the highest-possible resolution. Alternatively, you can convert the particles in your Dynamo table to a coordinates .star file ready for input into RELION 4.0 using the following tool: https://github.com/EuanPyle/dynamo2relion. Please check that the coordinates of the star file match expected particle positions.
